# A comprehensive review of Biomarkers associated with thyroid eye diseases

**DOI:** 10.22336/rjo.2026.52

**Published:** 2026

**Authors:** Divanshi Shrivastava, Ankita Aishwarya, Rachna Agarwal

**Affiliations:** 1 Department of Ophthalmology, Sanjay Gandhi PostGraduate Institute of Medical Sciences, Lucknow, Uttar Pradesh, India; 2 Department of Ophthalmology, PGICH, Noida, Uttar Pradesh, India

**Keywords:** thyroid eye disease, biomarkers, autoantibodies, microRNAs, precision medicine, TED = Thyroid Eye Disease, CAS = Clinical Activity Score, IGF1-R = Insulin-like Growth Factor 1 Receptor, IL-6 = Interleukin-6, IL6-R = Interleukin-6 Receptor, miRNA = MicroRNA, Th1 = T-helper cell type 1, TNF-α = Tumor Necrosis Factor-alpha, TRAb = Thyrotropin (TSH) Receptor Antibodies, TSI = Thyroid-Stimulating Immunoglobulins, TSHR = Thyroid-Stimulating Hormone Receptor, VISA = Vision, Inflammation, Strabismus, and Appearance

## Abstract

**Objective:**

Thyroid eye disease (TED), also known as Graves’ ophthalmopathy, is an autoimmune orbital disorder associated with Graves’ disease. Reliable biomarkers for early diagnosis, prognosis, and treatment monitoring remain limited. This review summarizes current evidence on biomarkers relevant to TED pathogenesis, progression, and clinical assessment.

**Methods:**

A comprehensive literature synthesis was conducted to evaluate reported serological, molecular, immunological, and genetic biomarkers implicated in TED, including autoantibodies, inflammatory cytokines, T-cell subsets, and microRNAs (miRNAs). Emerging omics-based and imaging-integrated biomarker discovery approaches were also examined.

**Results:**

Multiple biomarker classes show potential clinical utility for detecting disease activity, predicting progression, and assessing therapeutic response. Immunologic markers and circulating miRNAs demonstrate promise for early detection and risk stratification. Advances in high-throughput omics technologies and multimodal imaging are expanding the identification of candidate biomarkers. However, translation into clinical practice remains constrained by a lack of assay standardization, inconsistent cut-off values, heterogeneous study designs, and limited large longitudinal cohorts.

**Discussion:**

While current Thyroid Eye Disease (TED) assessments rely on subjective clinical scores, recent advances highlight the emerging utility of tear fluid cytokines (such as IL-6 and TNF-α) and molecular imaging markers to track active orbital inflammation and predict treatment outcomes. Integrating distinct serological, biochemical, and imaging biomarkers enables clinicians to transition to highly personalized, targeted therapeutic strategies.

**Conclusions:**

Biomarkers hold substantial potential to improve early diagnosis, prognostication, and personalized management of TED. Standardized validation studies and biomarker-stratified clinical trials integrated with omics and imaging platforms are essential to establish clinically reliable markers and enable precision-based care.

## Introduction

Thyroid eye disease (TED) is a complicated inflammatory condition that affects the orbit. It encompasses not only the orbital fibroblasts, adipocytes, and extraocular muscles but also the optic nerve and the orbital vasculature [1]. It is one of the most common causes of unilateral and bilateral proptosis in adults. About 25 to 40% of cases of Graves’ disease (GD) present with TED [2]. Systemic thyroid status can influence the progression of TED in an unpredictable manner [3].

TED is an autoimmune disorder characterized by inflammation and swelling of the orbital fat and extraocular muscles, occurring in a subset of individuals with Graves’ disease [4].

This disease affects orbital fibroblasts, with symptoms such as pain, diplopia, eyelid retraction, and exophthalmos. Under severe conditions, it can cause diminished vision, leading to functional and psychological challenges [5]. Women are often reported to be affected disproportionately, with an observed female-to-male ratio of 4.2:1, reflecting that there is a higher incidence of Graves’ disease in females [6]. Typically, TED develops closely in time with GD, and as many as 85% of patients experience eye symptoms within 18 months before or after the onset of thyroid dysfunction. It is also noteworthy that in up to 30% of cases, TED can precede the onset of hyperthyroidism [7].

While Thyroid Eye Disease clinically manifests in up to 40% of individuals with Graves’ disease, severe, sight-threatening manifestations impact only about 3%–5% of patients. There are two distinct age peaks at the outset, occurring in the fifth and seventh decades of life. Although the condition is more prevalent among women, the male-to-female ratio typically hovers around 1:1 up to 1:1.5, with severe forms of TED (**Fig. 1**).

**Fig. 1:**
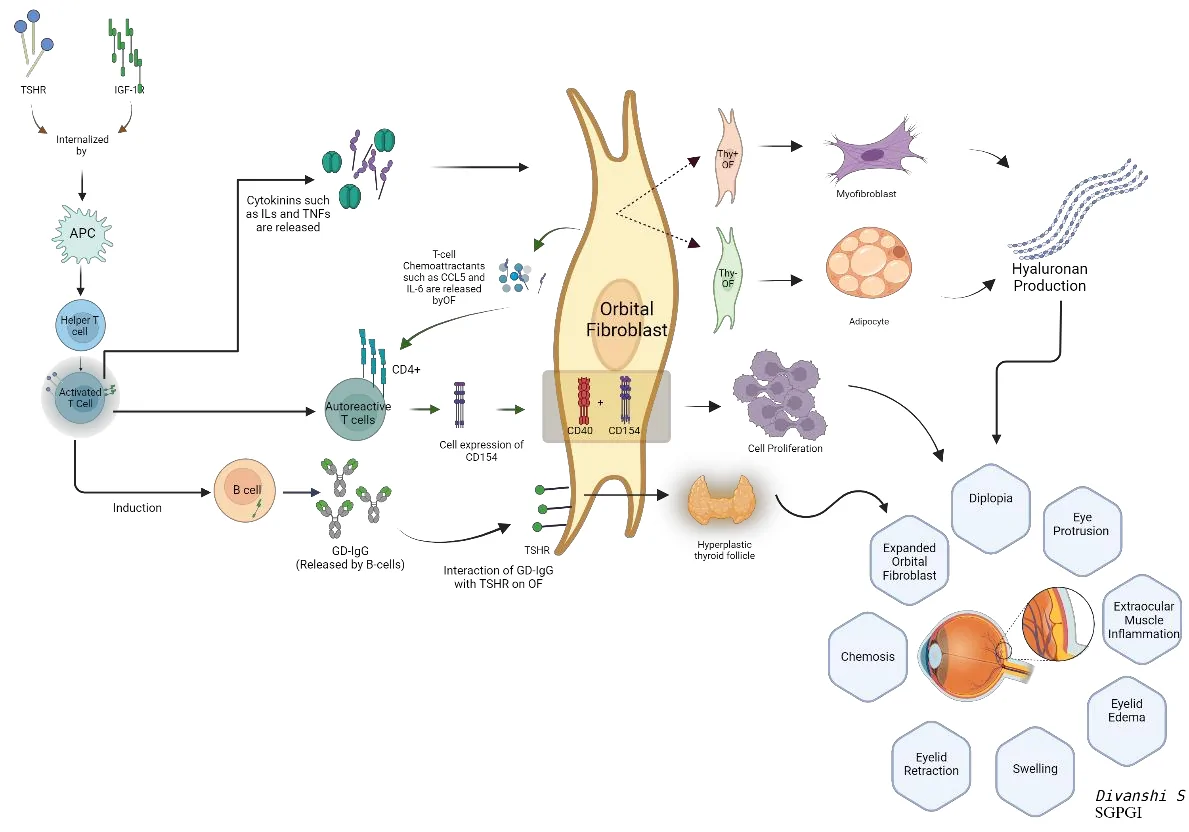
Pathogenic mechanisms driving Thyroid Eye Disease (TED). Schematic representation of the immunological and cellular interactions in TED, highlighting the role of TSHR, IGF-1R, orbital fibroblasts, and inflammatory mediators leading to tissue remodeling and clinical features such as proptosis, diplopia, and edema

Currently, Thyroid Eye Disease (TED) is not curable, and the therapeutic strategy primarily focuses on reducing inflammation and managing symptoms. According to the guidelines of the European Group of Graves’ Orbitopathy (EUGOGO), the initial treatment involves administering glucocorticosteroids [8]. However, glucocorticosteroid therapy is associated with a broad spectrum of adverse effects, and approximately 20% to 25% of patients do not respond to this treatment [9].

The identification of biomarkers sensitive and specific for Thyroid Eye Disease (TED) would be highly valuable, particularly in subclinical cases, where orbital manifestations precede thyroid dysfunction, and for monitoring treatment effectiveness. A biomarker is also essential because it can assist in assessing patients with Graves’ disease (GD) selected for radioiodine treatment, as predicting the development or worsening of TED in these patients is currently unreliable. For individuals deemed at risk of TED progression or development during radioiodine treatment, the European Group of Graves’ Orbitopathy (EUGOGO) guidelines recommend the initiation of oral prednisolone therapy, with a gradual reduction in the dosage over 3 months [2]. Furthermore, the discovery of novel biomarkers is likely to enhance our understanding of pathogenesis, opening new avenues for treatment [3].

Molecular biomarkers are biochemical molecules found in blood or other biological fluids, such as tears and urine, and in tissues that indicate the presence of an abnormal condition or disease [10,11]. In 1998, the National Institutes of Health Biomarkers Definitions Working Group defined a biomarker as “a characteristic that is objectively measured and assessed, as an indicator of normal biological and pathogenic processes, or pharmacological response to therapeutic interventions” [12].

Biomarkers span a wide range, from fundamental indicators like pulse and blood pressure to more complex laboratory tests involving blood and other tissues and biological fluids, such as tears. Medical signs, integral to clinical practice since the beginning of medicine, form the historical basis, while biomarkers represent the most objective and quantifiable evidence obtainable through modern laboratory science. The application of biomarkers is a relatively recent addition in clinical research, and optimal practices are still evolving in this domain [13]. Establishing the correlation between a measurable biomarker and pertinent clinical endpoints is a major challenge.

In medicine, biomarkers are organized by their clinical applications. These biomarkers can be primarily categorized as molecular, physiological, histological, and radiographic biomarkers [14].

Molecular biomarkers are detectable in body fluids or tissues, such as blood, urine, tears, or fat. These include genetic biomarkers such as DNA mutations, protein biomarkers such as antibodies, enzymes, and hormones, and metabolic biomarkers such as metabolites produced during metabolic processes [15].

A crucial preliminary step before developing a routine test is identifying the specific characteristics of the candidate molecular biomarker [16]. Various parameters are considered during this process, including the biomarker’s sensitivity and specificity for the disease, ease of access, accuracy, reproducibility, practicality, and ethical considerations.

In Thyroid Eye Disease, specific and sensitive biomarkers appropriately reflect the underlying immune-mediated responses, inflammation, adipogenesis, and tissue remodeling [7,8]. Biomarkers have emerged as essential tools not only for early detection and monitoring of the disease but also for personalized treatment. TED biomarkers can be classified into several categories based on their biological source and relevance [3,9]. In recent years, significant advances have been made in elucidating the molecular pathways underlying TED pathogenesis. This research has offered valuable insights into novel biomarker candidates.

For widespread clinical applicability and impact, biomarkers need to be readily obtainable without invasive procedures such as biopsy. Various molecules in blood, tears, and urine have been evaluated and suggested as potential biomarkers for Thyroid Eye Disease (TED) [3]. Most of the studies discussed in this article have employed conventional techniques such as ELISA, PCR, NTA, Western Blot, and cell-based assays to detect molecular biomarkers.

The focus of this review is to present an overview of the biomarkers discussed in the context of TED. This review highlights the significance of various biomarkers, including serological, cellular, metabolomic, epigenetic, and genetic biomarkers, in Thyroid Eye Disease.

## Materials and methods

We performed an intensive literature search and retrieved articles from PubMed, Embase, Scopus, and Web of Science up to July 2025 using keywords: “Thyroid Eye Disease”, “Graves’ orbitopathy”, “biomarkers”, “autoantibodies”, “cytokines”, “microRNA”, and “imaging”. In this review, we primarily included peer-reviewed original studies, RCTs, and translational research that report the diagnostic, prognostic, or therapeutic relevance of biomarkers in TED. We excluded case reports, editorials, conference abstracts, and studies that did not report a clear correlation between biomarkers and TED. Articles not published in English were also excluded. We included relevant clinical and translated studies in English.

We extracted data from the included articles based on study design, patient population, biomarker type, detection method, and biomarker clinical relevance. We narratively synthesized the findings and organized the review into a structured framework that classifies biomarkers as established, emerging, theranostic, or imaging (**Fig. 2**).

**Fig. 2:**
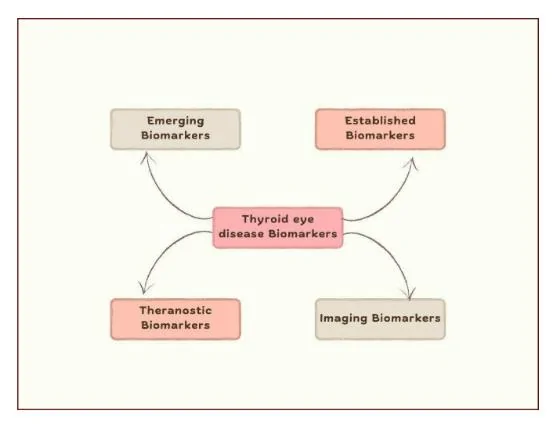
Types of biomarkers discussed in the article

This article broadly categorizes thyroid eye disease biomarkers into four types: emerging, established, theranostic, and imaging.

## Results

### Established Biomarkers

A wide range of biomarkers has been investigated in thyroid eye disease; some have been consistently validated and proven reliable and are already being utilized in clinical practice. These established biomarkers include TSHR antibodies (mainly TRAb and TSI), IGF-1R, TBII, and TPO-Ab.

Elevated TSI levels are the most common serological TED biomarkers [8]. These autoantibodies target the TSHR (thyroid-stimulating hormone receptor), leading to overproduction of thyroid hormone and stimulation of orbital fibroblasts [9].

Bartlena et al. reported that in Graves’ disease patients, high TSI levels might be a strong predictive factor of TED development, especially in smokers [10].

TRAb, which includes both stimulating and blocking antibodies directed against TSHR [7], is another established group of biomarkers that has been studied. Its persistent elevation after antithyroid treatment indicates ongoing autoimmune activity and has been correlated clinically with the CAS (clinical activity score) and a higher risk of Thyroid eye disease progression [16-18].

Autoantibodies directed against Insulin-like growth factor 1, which synergize with the TRAbs to stimulate cytokine production, adipogenesis, and fibroblast proliferation, are upregulated in TED [19].

Other serologically established TED biomarkers being used in clinical settings include TBII and TPO-Ab.

Proinflammatory cytokines and chemokines have also been important in the characterization of active TED, and some have been established as key players in the disease progression of Thyroid Eye Disease. The IL-6/IL-6R axis, known to suppress Treg differentiation and promote Th17 responses [20], is suggested to be elevated in orbital tissues and serum of TED patients and to correlate with disease activity.

The following table (**Table 1**) lists the key established biomarkers of TED and their roles in disease progression.

**Table 1:** List of Established Biomarkers of Thyroid Eye Disease and Their Clinical Relevance

Biomarker	Function/Role	Clinical Relevance in TAO
**TRAb** [1,2]	TSH receptor antibody	Elevated in TED; correlates with disease severity and CAS; predictive of GC response
**TSI** [3]	Thyroid-stimulating antibody (subset of TRAb)	Stimulates orbital fibroblasts and adipogenesis; associated with active TED
**TBII** [4]	TSH-binding inhibitory immunoglobulins	Detected in TED patients; higher levels in active disease
**TPO-Ab** [5,6]	Anti-thyroid peroxidase antibody	Elevated in autoimmune thyroid disease; weak association with TED severity
**IL-6/IL-6R** [21,22]	IL-6, IL-6Rα, gp130	Promotes Th17 differentiation, inflammation
**IGF-I/IGF-IR** [21-26]	IGF-I, IGF-IR, RANTES, IL-16	Fibroblast activation, cytokine secretion, hyaluronan synthesis
**CD40/CD154** [20]	CD40, CD154 (CD40L), ICAM-1, IL-6, PGE2	B cell activation, fibroblast proliferation, cytokine secretion

### Emerging Biomarkers

A growing number of studies have identified novel molecules involved in Thyroid Eye Disease that show strong potential as biomarkers beyond the established ones. The candidates include inflammatory cytokines, chemokines, microRNAs, and some other epigenetic regulators involved in tissue remodeling and underlying immune dysregulation in TED.

The most researched and top-signature emerging markers are cytokines, closely followed by chemokines. Several cytokines are directly involved in the pathogenesis of TED and thus can serve as valid biomarkers [16]. Elevations in serum levels of IL-1β, TNF-α, IFN-γ, and chemokines such as CXCL10 have been associated with active TED. These molecules are involved in the activation of orbital fibroblasts and leukocyte recruitment [17].

Studies have shown that CD40/CD154 costimulatory signaling is upregulated in orbital fibroblasts and T cells of patients with TED. This signaling drives the transcription of various inflammatory mediators, including ICAM-1 and prostaglandin E2, which are systemically elevated in patients with TED and may serve as biomarkers [18-20].

Another cytokine, TNF-α, which is central to the regulation of inflammation, is overexpressed in orbital fibroblasts from patients with TED. TNF-α polymorphisms are associated with disease severity and susceptibility [27].

Lower IL-1R levels have been observed in patients with TED. Members of the IL-1 superfamily, including IL-1α, IL-1β, IL-1Ra, IL-18, and IL-38 [28], have been shown to exhibit dysregulated expression in orbital fibroblast tissues from patients with TED [29].

Elevations in chemokines such as CXCL10, CXCL13, and RANTES in orbital fibroblasts and serum have been strongly associated with Th1-driven clinical activity and inflammation in TED [30].

Studies have reported decreased levels of cytokines and chemokines after corticosteroid and immunosuppressive therapy, supporting their significance as biomarkers of TED disease activity.

Recently, circulating microRNAs have emerged as promising, minimally invasive biomarkers for various autoimmune disorders, including TED, and have been reported to significantly influence gene expression, inflammation, fibrosis, and therapeutic response [31]. Distinct signature miRNAs, such as miR-146a, miR-155, miR-21, and miR-96, are dysregulated in orbital fibroblast tissues and PBMCs (peripheral blood mononuclear cells) of TED patients [32]. For instance, miR-146a normally suppresses NF-κB signaling, and its downregulation may exacerbate the inflammatory response [33]. miR-21 promotes fibrotic responses, while miR-155 enhances cell activation. miRNAs are biomarkers that effectively distinguish between the active and inactive phases of the disease [34].

**Table 2** presents key cytokine and chemokine biomarkers and their significance in the pathogenesis of TED.

**Table 2:** Key Emerging Biomarkers Implicated in TED Pathogenesis

Biomarker/Pathway	Sample/Sources	Expression in TAO	Functions in TAO
**CXCL10 (IP-10)** [11,12]	Chemokine induced by IFN-γ	Increased	An increase in TAO serum and tears correlates with disease activity
**IL-1 Superfamily** [16-18]	IL-1α, IL-1β, IL-1Ra, IL-18, IL-38	Increased	Pro- and anti-inflammatory regulation
**Chemokines** [29]	CXCL9/10/11, CXCL8, CXCL13, RANTES/CCL5	Increased	Immune cell trafficking, Th1 response
**miR-146a** [24,25]	Orbital tissue	Increased	Inhibits fibrosis via TGF-β signaling suppression
**TNF-α** [35]	TNF-α	Increased	Inflammation, ICAM-1 expression, GAG modulation, Elevated in active disease; promotes cytokine release and orbital edema
**miR-146a** [35-37]	Peripheral blood/CD4+ T cells	Decreased	Regulates immune response; inhibits Th1 differentiation
**miR-155** [37]	Peripheral blood	Increased	Promotes inflammation via TLR4/NF-κB; regulates CD4+ cytokines
**miR-21** [38,39]	Orbital fibroblasts	Increased	Promotes fibrosis and fibroblast proliferation
**miR-29 family (a/b/c)** [40]	Orbital fibroblasts	Decreased	Inhibits fibrosis via the Wnt/β-catenin pathway and collagen regulation
**miR-142-3p** [40]	Tregs (GPA)	Increased	Suppresses Treg function via the cAMP pathway
**miR-27a/b** [41]	Orbital fat tissue	Decreased	Inhibits adipogenesis via PPARγ and C/EBP suppression
**miR-224-5p** [42]	Serum	Lower in GC-insensitive cases	Predictive of glucocorticoid therapy response
**miR-96, miR-183** [43]	CD4+/CD8+ T cells	Increased after stimulation	Enhance T cell activation via Akt phosphorylation, suppress PTEN, and EGR-1
**miR-365, miR-148a** [43]	Orbital tissue (IOI)	Slightly increased	Linked to B-cell activity and IL-6 signaling in idiopathic orbital inflammation

Along with cytokines, chemokines, and microRNAs, some cellular markers have emerged as promising biosignatures of TED, with T-cell subsets being predominant amongst them. Previously, it has been hypothesized that T-cell-mediated immunity contributes to the development of thyroid eye disease. A thorough analysis of T cells, immune cell subsets, and their cytokine profiles can present crucial insights into the immunopathophysiology of TED [44].

T-cell subsets are central to TED pathogenesis. Previous studies have noted that T cells are responsible for B cell activation and autoantibody production. They produce cytokines and promote the expression of adhesion molecules [45]. Activated T cells, primarily CD4+ T cells, produce various adhesion molecules. Cytokines produced by the CD4+ T cells maintain and amplify orbital inflammation and aggravate the immune response in Thyroid Eye Disease [46]. CD4+ T helper cells play a leading role in cellular immunity and contribute to humoral immunity. They differentiate into functionally distinct subsets, such as Th1, Th2, Th17, and Treg cells, which play significant roles in immunity and autoimmunity [47].

Th1 cells mediate cellular immunity and produce inflammatory cytokines. They are elevated in TED, and their cytokines correlate with disease activity and tissue inflammation [48].

Th2 cells support humoral immunity by stimulating B cells. During the later stages of TED, Th2 cells predominantly infiltrate orbital fibroblasts, suggesting a shift from a cell-mediated immune response to a humoral response [49,50].

Another class of T helper cells, the Th17 cells, is defined by the secretion of IL-17A. Studies have shown that Th17 cells are involved in many autoimmune diseases. Expression of Th17 cells and IL-17A is elevated in peripheral blood and tears, promoting inflammation and fibrosis in orbital fibroblasts (OFs) in TED [51]. The interaction between Th17 cells and CD34+ fibrocytes amplifies local inflammation [52].

Regulatory T (Treg) cells, such as CD4+, CD25+, and FOXP3+, are involved in regulating immune responses. Dysfunction or altered levels of Treg cells have been linked to TED severity. Studies show that decreased CTLA-4 mRNA and increased FOXP3 mRNA levels suggest disrupted Treg function in severe cases of TED [53,54].

Although emerging evidence suggests a role for Th22 cells in active TED, the pathogenic significance of Th9 cells and the contributions of both Th9 and Tfh subsets to disease progression remain insufficiently characterized [55].

An overview of the T cell subsets and their relevance as emerging biomarkers of TED is provided in **Table 3**.

**Table 3:** T Cell Subset Alterations and Their Potential Role as Biomarkers in TED

T Cell Subset	Key Markers	Functions	Findings in TED
**Th2 (CD4⁺)** [48]	IL-4, IL-5, IL-13	Drives humoral immunity, B-cell activation	↑ In late/chronic TED, dominant in long-duration disease
**Th17 (CD4⁺)** [19,51,52]	IL-17A, IL-17F, IL-22	Promotes inflammation, autoimmunity	↑ IL-17A in blood, tears, and orbital tissues promotes fibrosis and inflammation of OFs
**Tfh (CD4⁺)** [55]	IL-21, Bcl6	Supports B-cell differentiation in follicles	Not well studied in TED
**Th9 (CD4⁺)** [51]	IL-9	Involved in hypersensitivity, skin disorders	Limited evidence in TED, located in peripheral blood and skin
**Th1 (CD4⁺)** [48,50]	IFN-γ, IL-2, TNF-α	Mediates cellular immunity, delayed hypersensitivity	↑ in early TED; correlates with inflammatory activity; dominates in muscle tissue
**Th22 (CD4⁺)** [51]	IL-22	Infiltrates the inflamed dermis	IL-22 ↑ in other dermatoses; role in TED unclear
**Treg (CD4⁺CD25⁺FOXP3⁺)** [53,54]	TGF-β, IL-10, CTLA-4	Suppresses immune responses; maintains tolerance	↓/dysfunction associated with TED severity; ↑ post-treatment; CTLA-4 ↓ in severe TED
**CD8⁺ T Cells** (Tc1, Tc2, Tc17, CD8⁺ Tregs) [6,16,55]	IFN-γ, IL-5, IL-9, IL-17, TGF-β	Cytotoxicity, immune regulation	↑ CD4/CD8 ratio in TED; ↓ CD8⁺ in early TED/GD; CD8⁺ T cells produce Th1/Th2 cytokines in situ
**γδ T Cells** [40]	IL-17A	Innate-like T cells with regulatory and proinflammatory roles	Predominant in orbital tissues; source of IL-17A

### Theranostic Biomarkers

Another exciting subset of TED biomarkers comprises molecules that not only reflect disease pathophysiology but also serve as excellent therapeutic targets. These types of biomarkers are pivotal bridges between diagnosis and treatment. Keystone theranostic biomarkers identified so far include IGF-1R, IL-6/IL-10R, and TNF-alpha.

IGF-1R, the Insulin-like growth factor 1 receptor, displays a close partnership with TSHR, the thyroid-stimulating hormone receptor, and the two receptors together form a functional alliance that is responsible for amplifying the immune response, leading to proliferation of fibroblasts, fat accumulation, hyaluronan production, and swelling, ultimately resulting in tissue expansion in the orbit [56].

Teprotumumab, a blocking monoclonal antibody against IGF1-R, is being used as a therapeutic agent in TED, underscoring the dual relevance of IGF-1R antibodies as both an established biomarker and a therapeutic target [57].

Similarly, Tocilizumab (TCZ), an IL-6R monoclonal antibody, has shown efficacy in patients with steroid-resistant TED [58,59]. By interrupting the IL6/IL6-R signaling, this weakens Th17 signals and responses, restoring a degree of balance and relieving inflammation.

Anti-TNF-α agents, being widely used in other autoimmune disorders, have also produced promising results in TED so far. Higher concentrations of TNF-α in orbital tissues and serum have been correlated with disease activity and severity [35].

While it is not yet part of mainstream therapy, targeting TNF-α could provide a way to monitor and treat the disease. TNF-α seems promising as an emerging therapeutic biomarker.

In TED, increased IL6 levels have been associated with worsening tissue damage and increased orbital swelling, which is why the IL6/IL6-R axis emerges as a key stone involved in the suppression of Treg differentiation and the promotion of the Th17 response. Research shows that IL6 and its receptors are elevated in both serum and orbital tissue of TED patients and are closely associated with disease activity and severity [60].

### Imagining Biomarkers

In addition to the various established and emerging serological, cellular, epigenetic, and molecular biomarkers and therapeutic targets discussed in this review so far, imaging technologies have gained attention for their ability to provide reproducible, objective TED biomarkers. Quantitative parameters derived from imaging technologies such as CT, MRI, and OCT have provided meaningful insights into the disease activity and severity of TED and have also supported molecular approaches. Recent studies have regarded quantitative imaging as a valuable approach to identifying structural biomarkers in thyroid eye disease [61,62].

A study by Chaganti et al. in 2022 employed multi-atlas automated segmentation of orbital CT scans and identified two major imaging phenotypes: a stretched optic nerve phenotype with a longer optic nerve and increased orbital fat, and a large-volume phenotype with enlarged extraocular muscles. Both phenotypes correlate clinically with distinct features of thyroid eye disease, such as restricted motility, vision loss, and proptosis [63].

Other studies have also complemented this approach by using CT-based measurements of Thyroid Eye Disease. An increase in volumes of the individual rectus muscles significantly predicted the risk of dysthyroid optic neuropathy [64,65]. Apex crowding indices on CT imaging have high specificity and sensitivity for DON identification [66]. In addition to CT, radiomics from OCT (optical coherence tomography) has been used to distinguish active from inactive TED using machine-learning classifiers. Studies have explored diffusion-weighted MRI to assess posterior orbital involvement in TED [67,68].

These studies demonstrate that the role of imaging biomarkers, especially CT-based quantitative metrics, has been evolving for TED. These biomarkers can be valuable in enhancing phenotyping, precise diagnosis, risk assessment, and stratification in TED. The findings have been summarized in **Table 4**.

**Table 4:** Summary of Studies Investigating Imaging Biomarkers in Thyroid Eye Disease (TED)

Study	Imaging Modality & Method	Key Findings
Chaganti et al. (2022) [63]	CT with automated multiatlas segmentation and PCA	Identified two phenotypes: big muscle volume (associated with motility loss) and stretched optic nerve (associated with proptosis and smoking)
Gonçalves et al. (2012) [64]	CT-based apex crowding index	High sensitivity and specificity of the apex crowding index for DON diagnosis
Ma et al. (2024) [65]	OCT radiomics with machine learning	Differentiated active vs. inactive TED with AUC ~0.84
Alkhadrawi et al. (2024) [67]	CT with deep learning CNN	Automatic detection of extraocular muscle enlargement in TED
Potvin et al. (2025) [68]	Diffusion-weighted MRI	Quantified posterior orbital involvement

Flowchart demonstrating the role of various biomarkers in thyroid eye disease across diagnosis, risk stratification, disease activity assessment, therapeutic decision-making, and post-treatment monitoring (**Fig. 3**).

**Fig. 3:**
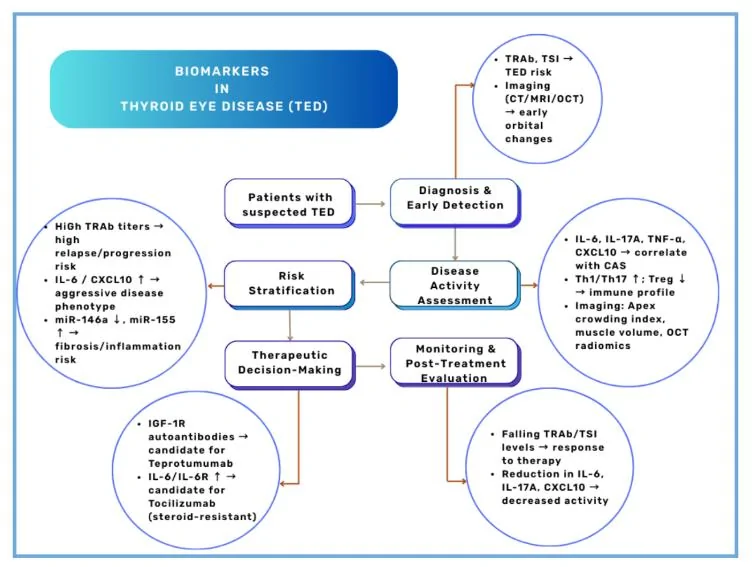
Role of biomarkers in thyroid eye disease

## Discussion

Our review highlights the importance of biomarkers as a diagnostic, monitoring, and even therapeutic tool for TED. Through this comprehensive literature search, we found that biomarkers offer a valuable opportunity to refine clinical practice by improving diagnosis and monitoring, enabling risk stratification, and guiding treatment. Currently, for TED assessment, disease activity and severity are primarily analyzed using clinical activity scores such as CAS and VISA, which are valuable but limited by subjectivity and interrater variability. Biomarkers have significant potential to improve current clinical approaches to TED assessment and management.

At the diagnostic stage, various serological, protein, and cellular biomarkers, such as IL-6, TNF-α, TRAb, TSI, and specific microRNAs, can be appropriately used to provide more objective and quantifiable measures of disease activity and severity. For instance, elevated IL6/IL6-R levels have been correlated with disease activity in studies and can help identify patients most likely to respond to Tocilizumab, which blocks IL6. Similarly, anti-TNF therapy could be beneficial for patients with elevated TNF-α and Th1 chemokine levels. Biomarkers involved in the IGF1-R/TSHR pathway activation have been linked with adipogenesis and remodeling of orbital tissues, making them relevant for selecting patients who can benefit from Teprotumumab therapy.

Evaluating biomarker levels over time can also enable early relapse detection, treatment response monitoring, and even the identification of the safest window for surgical interventions in severe-to-life-threatening cases.

Thus, our review emphasizes that biomarkers can be systematically incorporated into clinical practice, enabling classification of patients into inflammation-dominant or remodeling-dominant phenotypes using an organized diagnostic panel, with baseline biomarker levels guiding initial therapy selection. Over-time measurements can support monitoring of disease progression, the effects of therapy and medications, and the risk of relapse.

However, to translate this promise into practice, there is an urgent need for well-designed, high-quality prospective studies. Longitudinal cohort studies can establish standardized assays, cut-off values, and correlations between biomarker levels and CAS, imaging, and patient outcomes. Large longitudinal cohort studies would help validate reproducibility across serum, plasma, and tear samples; define clinically relevant threshold values that can be correlated with disease stages, activity, and severity, as well as treatment responses; and provide long-term outcomes.

Biomarker-stratified randomized trials are required to provide high-level evidence on whether selecting therapies based on cytokine profiling yields higher efficacy than conventional approaches.

Moreover, composite indices that integrate cytokines, chemokines, autoantibodies, and miRNAs with CAS and orbital imaging would greatly help enhance precision, as they encompass both inflammatory and remodeling pathways.

Pragmatic implementation studies and health economic evaluation can be conducted in parallel, as they are critical for establishing feasibility, validating cost-effectiveness, and assessing scalability across different clinical settings.

## Conclusion

Ultimately, our search has led us to believe that with intensive research and proper validation, biomarker-guided algorithms can enable an earlier, more accurate prognosis and diagnosis of high-risk patients, help in tailoring therapy according to the underlying disease pathophysiology, avoid unnecessary toxicity, reduce long-term morbidity, and transform Thyroid Eye Disease management significantly.
